# Sinularin Induces Oxidative Stress-Mediated Apoptosis and Mitochondrial Dysfunction, and Inhibits Angiogenesis in Glioblastoma Cells

**DOI:** 10.3390/antiox11081433

**Published:** 2022-07-23

**Authors:** Shih-Yuan Hsu, Zhi-Hong Wen, Po-Chang Shih, Hsiao-Mei Kuo, Sung-Chun Lin, Hsin-Tzu Liu, Yi-Hsin Lee, Yi-Jen Wang, Wu-Fu Chen, Nan-Fu Chen

**Affiliations:** 1 Department of Marine Biotechnology and Resources, National Sun Yat-sen University, Kaohsiung 80424, Taiwan; 07077@ptch.org.tw (S.-Y.H.); wzh@mail.nsysu.edu.tw (Z.-H.W.); po-chang.shih.14@ucl.ac.uk (P.-C.S.); hsiaomeikuo@gmail.com (H.-M.K.); lesinsin@gmail.com (Y.-H.L.); yijenwang0217@gmail.com (Y.-J.W.); ma4949@cgmh.org.tw (W.-F.C.); 2 Department of Neurosurgery, Pingtung Christian Hospital, Pingtung 90059, Taiwan; 3 Department of Neurosurgery, Kaohsiung Chang Gung Memorial Hospital and Chang Gung University College of Medicine, Kaohsiung 83301, Taiwan; 4 Center for Neuroscience, National Sun Yat-sen University, Kaohsiung 80424, Taiwan; 5 Department of Orthopedic Surgery, Pingtung Christian Hospital, Pingtung 90059, Taiwan; linsungchun@yahoo.com.tw; 6 Department of Medical Research, Hualien Tzu Chi Hospital, Buddhist Tzu Chi Medical Foundation, Hualien 970473, Taiwan; hsintzuliu@tzuchi.com.tw; 7 Division of Neurosurgery, Department of Surgery, Kaohsiung Armed Forces General Hospital, Kaohsiung 80284, Taiwan; 8 Institute of Medical Science and Technology, National Sun Yat-sen University, Kaohsiung 804201, Taiwan; 9 Center for General Education, Cheng Shiu University, Kaohsiung 833301, Taiwan

**Keywords:** GBM, oxidative stress, mitochondrial dysfunction, angiogenesis, zebrafish, natural product, redox-active compounds

## Abstract

Glioblastoma multiforme (GBM) is a cancer of largely unknown cause that leads to a 5-year survival rate of approximately 7% in the United States. Current treatment strategies are not effective, indicating a strong need for the development of novel therapies. In this study, the outcomes of sinularin, a marine-derived product, were evaluated against GBM. Our cellular studies using GBM cells revealed that sinularin induces cell death. The measured half maximal inhibitory concentrations (IC_50_) values ranged from 30 to 6 μM at 24–72 h. Cell death was induced via the generation of ROS leading to mitochondria-mediated apoptosis. This was evidenced by annexin V/propidium iodine staining and an upregulation of cleaved forms of the pro-apoptotic proteins caspase 9, 3, and PARP, and supported by CellROX^TM^ Green, MitoSOX^TM^ Red, and CM-H_2_DCFDA staining methods. In addition, we observed a downregulation of the antioxidant enzymes SOD1/2 and thioredoxin. Upon treatment with sinularin at the ~IC_50_ concentration, mitochondrial respiration capacities were significantly reduced, as shown by measuring the oxygen consumption rates and enzymatic complexes of oxidative phosphorylation. Intriguingly, sinularin significantly inhibited indicators of angiogenesis such as vessel tube formation, cell migration, and cell mobility in human umbilical vein endothelial cells or the fusion cell line EA.Hy926. Lastly, in a transgenic zebrafish model, intersegmental vessel formation was also significantly inhibited by sinularin treatment. These findings indicate that sinularin exerts anti-brain cancer properties that include apoptosis induction but also antiangiogenesis.

## 1. Introduction

The most aggressive type of brain cancer is glioblastoma multiforme (GBM), which is categorized as grade IV and accounts for approximately half of all glioma types. The incidence of GBM is higher in males than females and in Caucasians compared to other ethnicities. This astrocytic-type brain tumor was reported to arise de novo in >90% of patients [1]. In addition to its high aggressiveness, the highly infiltrative nature of GBM results in low responses to available treatments. Clinically, GBM disease is refractory to conventional therapies, including the standard chemotherapeutic drug temozolomide (TMZ) agent, but also surgical resection, leading to a median survival of 12–15 months and a 5-year survival rate of approximately 7% in the United States [2]. Developing new therapies against GBM is, thus, a pressing matter.

Mitochondria are actively involved in metabolic reprogramming with an enriched concentration of reactive oxygen species (ROS). Studies have reported that anti-cancer agents can disrupt mitochondrial functions via increased ROS, impaired oxidative phosphorylation (OXPHOS), exacerbated extracellular acidification rate, and the degradation of mitochondria (also known as mitophagy). Moreover, the damage to mitochondria by anti-cancer agents can lead to an activation of intrinsic apoptosis pathways. To lessen excessive ROS-induced cell damage and apoptosis, cells utilize intrinsic antioxidant enzymes, such as superoxide dismutase (SOD) and thioredoxin, to reduce oxidative stress, hence abrogating cytotoxicity [3].

Angiogenesis is a process by which new blood vessels are formed from existing ones. This formation is crucial for embryonic and fetal development as well as organ growth, and it supports wound healing [4,5]. Angiogenesis can be initiated by a lack of oxygen within the tissue or organ [6]. Angiogenesis is tightly regulated by the balance between pro- and antiangiogenic signals, such as integrins, chemokines, angiopoietins, oxygen sensing agents, and endogenous inhibitors [7]. Intriguingly, angiogenesis is a hallmark of over 50 different disease states, while its dysfunction is implicated in multiple disorders, including cancer [8]. Typically, when tumors exceed 2 mm in diameter, passive diffusion becomes inadequate in providing nutrients. Cell proliferation is then counterbalanced by cell death. Hence, to grow further, the tumor has to become angiogenic by expressing pro-angiogenic factors such as vascular endothelial growth factor (VEGF), thereby changing the equilibrium between pro- and antiangiogenic mediators, a process known as an angiogenic switch [9]. Antiangiogenesis has accordingly become a key strategy to prevent tumor growth.

Approximately 78% of anti-cancer agents are structurally derived from natural products [10]. The oceans account for nearly 70% of the total world area, while the diversity of marine ecosystems such as coral reefs is much higher compared to tropical rainforests. This has made the oceans a treasure trove to acquire novel compounds for drug development. However, marine-derived compounds have been less investigated compared to terrestrial ones [11]. Many metabolites extracted from sea creatures were shown to present significant anti-inflammatory and anti-cancer effects. Sinularin is a natural product that can be sourced from the soft coral *Sinularia flexibilis*. This compound possesses antineoplastic activities against human epidermoid carcinoma, P388 lymphocytic leukemia, and oral, hepatocellular, gastric, breast, renal, and melanoma cancers [12,13,14,15,16]. However, the effects of sinularin on angiogenesis remain to be clarified. In the present study, we evaluated the effects of sinularin on angiogenesis, in addition to cell viability and mitochondrial functions, in brain cancer cells.

## 2. Materials and Methods

### 2.1. Reagents

A stock solution of sinularin was prepared in dimethyl sulfoxide (DMSO), protected from light, and stored at −20 °C. The tetrazolium reagent 3-(4,5-di-methylthiazol)-2,5-diphenyltetrazolium bromide (MTT) was purchased from Sigma-Aldrich Chemical Co. (St. Louis, MO, USA).

### 2.2. Cell Culture

GBM 8401 (BCRC, CVCL_B051), U87 MG (ATCC^®^ HTB14), T98G (ATCC^®^ CRL-1690), and U138 MG (ATCC^®^ HTB-16) were the commercial GBM cell lines of Bioresource Collection and Research Center (Hsinchu, Taiwan) or American Type Culture Collection (Manassas, VA, USA). Alpha-MEM medium (Gibco BRL, Rockville, MD, USA) was used for the maintenance of U87 MG, T98G, and U138 MG, while GBM 8401 cells were cultured in RPMI-1640 medium (Gibco). These cells were supplemented with 10% fetal bovine serum, 100 U/mL penicillin, and 100 µg/mL streptomycin (Gibco) and cultured under a humidified atmosphere of 5% CO_2_ and 95% room air at 37 °C. EA.hy926 cells (ATCC^®^ CRL2922™) was an established human fusion cell line by fusing primary human umbilical vein cells with a thioguanine-resistant clone A549. The fusion cells were cultured in Dulbecco’s Modified Eagle’s Medium with 10% of fetal bovine serum and 100 U/mL of penicillin and streptomycin and cultured under the same atmosphere as for the GBM cells.

### 2.3. Annexin V-FITC/Propidium Iodide Staining

The cells were treated with sinularin at various concentrations for 24 h and the cells were harvested, washed with cold PBS, and re-suspended in 1× binding buffer to make a concentration of 6 × 10^5^ cells/mL. A number of cells (6 × 10^4^) were then transferred to a 5 mL culture tube. All samples were processed for Annexin V label according to the literature [17,18]. At the end of the incubation, 1 mL of 1× binding buffer was added to each sample and the sample was analyzed using a flow cytometer Beckman Coulter cytometer (Beckman-Coulter, Brea, MI, USA) with the use of Cell Lab Quanta™ SC analysis software. A minimum of 10,000 cells per sample were analyzed.

### 2.4. Detection of ROS

Intracellular ROS was evaluated by a CM-H2DCFDA fluorescent probe. Mitochondrial superoxide (O_2_^•−^) level was quantified by MitoSOX^TM^ Red (Molecular Probes, Inc., Eugene, OR, USA). CellROX^®^ Green Reagent was applied to measure oxidative stress in live cells. The procedures for each staining method were conducted as described in the literature [17]. The fluorescence yielded from the staining reagents was detected using a Beckman Coulter cytometer (Beckman-Coulter, MI, USA) with the use of Cell Lab QuantaTM SC analysis software. A minimum of 10,000 cells per sample were analyzed.

### 2.5. Mitochondrial Oxygen Consumption Measurements

Mitochondrial oxygen consumption measurements were assessed as described in the literature [17]. Briefly, a Seahorse XF24 Extracellular Flux Analyzer from Seahorse Bioscience (Chicopee, MA, USA) was acquired for detecting consumption of oxygen in mitochondria. The GBM cells were seeded in 24-well plates at 1 × 10^5^ cells/well treated with specified media and placed for 16–18 h in the 37 °C incubator (5% CO_2_ and 95% air). This density number reached about 90% confluence. The culture media were then replaced with sinularin-containing medium for 24 h of incubation. In a 24 micro-well calibration plate was added 675 μL calibration solution for background correction; however, the software would automatically subtract the background values after the experimental analysis was completed. The cells were washed with 0.5 mL of DMEM without sodium bicarbonate, pH = 7.4, and each well was replenished with 675 μL of DMEM for further examination. Four measurements of the basal OCR were averaged under basal conditions, followed by treating cells sequentially with 1 μM oligomycin, 250 nM of FCCP, and 2 μM of rotenone. A standard curve of protein concentration was generated using bovine serum albumin with a DC protein assay kit (Bio-Rad, Hercules, CA, USA). To compare results, data were calculated after normalizing with the protein concentration.

### 2.6. Cell Proliferation Assay

Cell viability levels were assessed using an MTT staining assay following treatment with various concentrations of sinularin for 24–72 h. The MTT drug was added to each well, and the plates were incubated at 37 °C for 2–4 h to allow MTT reduction by the enzyme dehydrogenase of live cells, resulting in media-insoluble formazan. Briefly, the cells were plated in triplicate at a density of 3 × 10^4^ cells per well in 96-well plates (Nunc, Roskilde, Denmark). Following overnight incubation, the cells were treated with sinularin at concentrations of 0–100 μΜ for 24–72 h. Absorbance was measured at 570 nm using an ELISA reader (Dynatech Laboratories, Chantilly, VA, USA).

### 2.7. Wound Healing Assay

The wound healing assay is a typical test that allows the observation of cell migration. The degree of migration was determined by the speed and amount of cell growth. Cells were grown until 90% confluence, followed by drawing several lines with a pipette tip. The cells were treated with sinularin at 0–10 μΜ, then incubated for 20 h. Using a microscope to capture pictures, the degree of cell growth at the edge of the line was observed.

### 2.8. Transwell Chamber Migration Assay

The migration abilities of EA.hy926 cells following sinularin treatment were evaluated in triplicate using the transwell chamber migration assay. Transwell inserts with 8-μm pore size (Corning Inc., Corning, NY, USA) were selected for this assay. Endothelial cells were seeded on top of the filter membrane at a density of 2 × 10^4^ cells/chamber using 1% FBS containing the specified sinularin concentrations. The lower chamber was filled with 10% FBS as a chemo-attractant to induce cell migration. Following 24 h of incubation, with care, cotton-tipped applicators were used to remove residual solution and remaining cells from the upper part of the membrane. Migrated cells on the other side of the membrane were washed with 1× PBS, fixed using 4% paraformaldehyde, and 10% Giemsa-stained for 25 min. A phase-contrast microscope (Leica Microsystems, Wetzlar, Germany) was utilized for observations of the lower part of membrane, and the images were captured using a SPOT CCD RT-slider integrating camera (Diagnostic Instruments, Sterling Heights, MI, USA). Transwell migration was analyzed on the captured images with the ImageJ analysis software, which allowed us to evaluate the number of migrated cells within three randomly selected regions on each transwell insert.

### 2.9. Tube Formation Assay

The tube formation assay was performed as previously described (Kuo et al., 2012). In brief, Matrigel (Becton Dickinson; Bedford, MA, USA) was diluted with cold serum-free medium to 10 mg/mL. A volume of 70 μL of this diluted Matrigel solution was added to each well in 96-well plates, followed by incubation at 37 °C for 30 min to allow for the formation of a gel. A solution of 100 μL of the cell suspension (3 × 10^4^ cells) using 10% FBS containing specified sinularin concentrations was then subsequently added to each well and incubated for 6–8 h at 37 °C, within a 5% CO_2_ and 95% room air environment. Under these conditions, endothelial cells formed delicate networks of tubes that were detectable within 2–3 h and were fully developed after 8–12 h. After incubation, the endothelial cell-constituted tubes were fixed with 3% paraformaldehyde and counted under four different high-powered fields.

### 2.10. Western Blot Analysis

Cells were lysed and their proteins dissolved in a protein extraction reagent (Thermo Scientific, Waltham, MA, USA). Information on the primary antibodies against the studied proteins is summarized in Supplementary File Table S1. Following conjugation of the secondary antibody with horseradish peroxidase at 37 °C for 1 h, the signals on the membrane were detected by enhanced chemiluminescence (ECL-kit; Millipore, Burlington, MA, USA). Photos were taken after visualization of the protein bands using a UVP BioChemi imaging system (UVP LLC, Upland, CA, USA). Relative densitometry quantification of the bands was performed using the LabWorks 4.0 software (UVP LLC). A β-actin antibody was used to reprobe the PVDF membranes as a loading control.

### 2.11. Zebrafish Breeding and Embryo Generation

The animal studies were conducted following the National Research Council’s Guide for the Care and Use of Laboratory Animals. The zebrafish were reared in a five-layer self-contained zebrafish culture system (Taigang Technology). The photoperiod, daily feeding, and maintenance of water quality were ensured. One female and two males were separated on both sides of a transparent partition in the paired tank, and the partition was removed for two hours before the start of the dark cycle. The next day, after light stimulation, the zebrafishes started tail-chasing, spawning, and fertilization. Then, the fertilized eggs under the pairing tank were collected and transferred to a culture medium containing Hank’s buffer (100 mL of deionized water with 20 mg KCl, 142 mg Na_2_HPO_4_, 24 mg KH_2_PO_4_, 800 mg NaCl, 17.5 mg NaHCO_3_, 72 mg CaCl_2_, and 123 mg MgSO_4_). The dish was placed at 28 °C in a lighted incubator.

### 2.12. Drug Treatment in Zebrafish and Intersegmental Angiogenesis

At one day postfertilization, the zebrafish fertilized eggs were divided into groups, and sinularin was dissolved into Hank’s buffer. The control group received the same amount of DMSO as the test substance. The treated and control groups then returned to the box at 28 °C for culture under light condition. On the second day of fertilization, the zebrafish fertilized eggs began to hatch larvae. The larvae were subsequently immersed in MS-222 (168 ppm tricaine mesylate) for anesthesia and placed on a depression slide. The formation of intersegmental blood vessels in the zebrafishes was photographed (Leica DMI 6000B). For quantification, the ImageJ software was used to select the area of intersegmental blood vessels on the back of each fish and calculate the average fluorescence intensity of the area.

### 2.13. Statistical Analysis

The SPSS software (Windows 13.0 version; SPSS Inc., Chicago, IL, USA) was used for all statistical analyses. IC_50_ values were determined using a four-parameter logistic function. Results were analyzed using a Student’s *t*-test and presented as the mean ± standard error of the mean. * *p* < 0.01 or ** *p* < 0.05 were considered as statistically significant.

## 3. Results

### 3.1. Sinularin Induces Different Levels of Cytotoxicity in Four Brain Cancer Cell Lines

**Figure 1:**
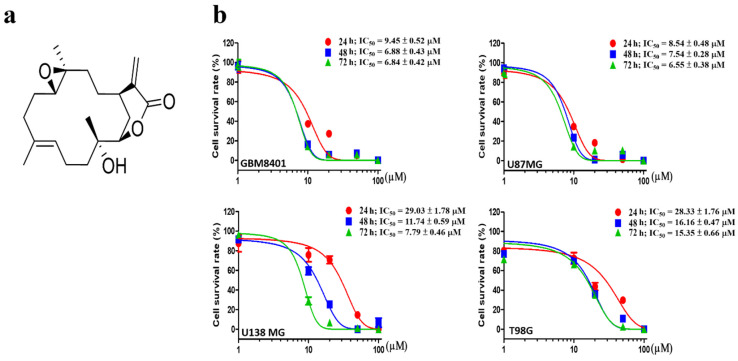
The effects of sinularin on brain cancer cell viability. (**a**) Chemical structure of sinularin. (**b**) Concentration–response curves of cell viability for GBM 8401, U87 MG, U138 MG, and T98G after 24, 48, and 72 h of sinularin treatment as well as determined IC_50_ values. Each bar represents the mean ± standard error of the mean.

Four different brain cancer cell lines, U87 MG, GBM 8401, U138 MG, and T98G, were used to assess cell viability under sinularin treatment for 24, 48, or 72 h with a range of concentrations (0–100 μM) (Figure 1a). The half maximal inhibitory concentration (IC_50_) values of sinularin were 8–30 μM, 7–16.5 μM, and 6–16 μM after incubation for 24, 48, and 72 h, respectively (Figure 1b). The cell line U87 MG was generally more sensitive to sinularin compared to the other cell lines, while T98G was the least vulnerable. Subsequently, a lower range of 0–20 μM, with the highest concentration corresponding to the IC_50_ values, was used to study the impact of sinularin on the most sensitive cell line, U87 MG, in the following studies.

### 3.2. Sinularin Triggers Apoptosis and DNA Damage via Activation of Caspase 9/3 and PARP

**Figure 2:**
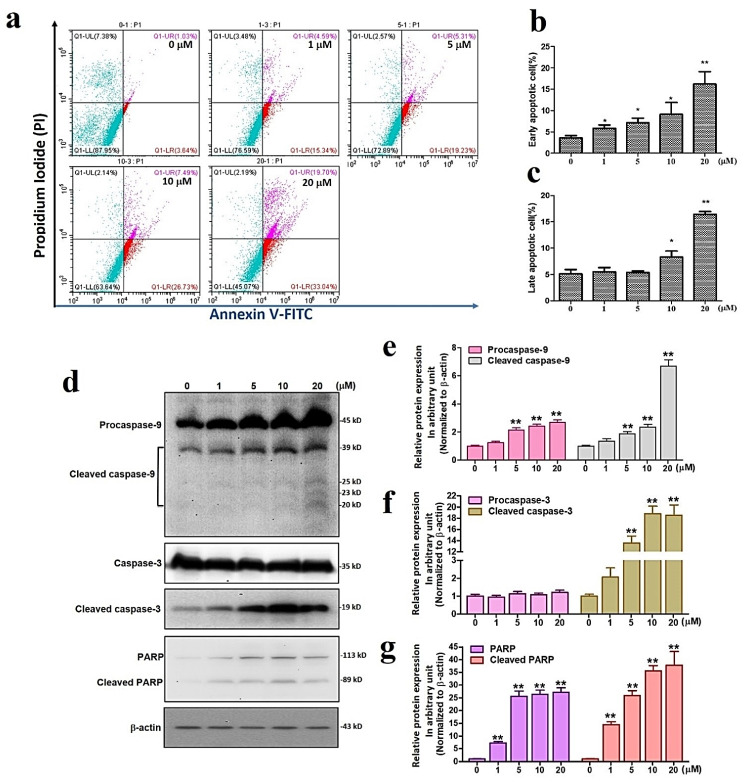
The effects of sinularin on apoptosis and DNA damage. (**a**) Annexin V/propidium iodine staining chart showing the effects of sinularin at 0–20 μM on the death of U87 MG cells. (**b**) Analysis of early apoptosis. (**c**) Analysis of late apoptosis. (**d**) Western blot measuring the protein levels of caspase 9/3 and PARP and their cleaved forms. Full, uncropped Western blot gel can be found on Supplementary File Figure S1a. (**e**) Analysis of caspase 9 and its cleaved form. (**f**) Analysis of caspase 3 and its cleaved form. (**g**) Analysis of PARP and its cleaved form. Each bar represents the mean ± standard error of the mean. * *p* < 0.05 and ** *p* < 0.01 relative to control.

We subsequently analyzed the proportion of apoptotic cells resulting from sinularin in U87 MG cells. The annexin V/propidium iodine (PI) staining method was used to assess early and late apoptosis. In the annexin V/PI chart, the upper right quadrant identifies a population of late apoptotic cells, while the lower right quadrant indicates the early apoptotic cells. Our findings show that early apoptotic cells were significantly increased at 1 μM of sinularin and above concentrations, whereas a significant increase was observed at 10 μM and above concentrations (Figure 2a–c). Considering these increased numbers of early and late apoptotic cells, we subsequently evaluated the apoptosis markers caspase 9/3 and the DNA damage marker poly ADP-ribose polymerase (PARP). Our results reveal that the expression levels of cleaved caspase 9/3 and PARP were significantly increased with 5 μM of sinularin and above concentrations (Figure 2d–g). Together, these findings indicate sinularin-induced apoptosis and DNA damage via the activation of caspase 9/3 and PARP.

### 3.3. Sinularin Significantly Elevates the Levels of ROS and Reduces Antioxidant Protein Activities

**Figure 3:**
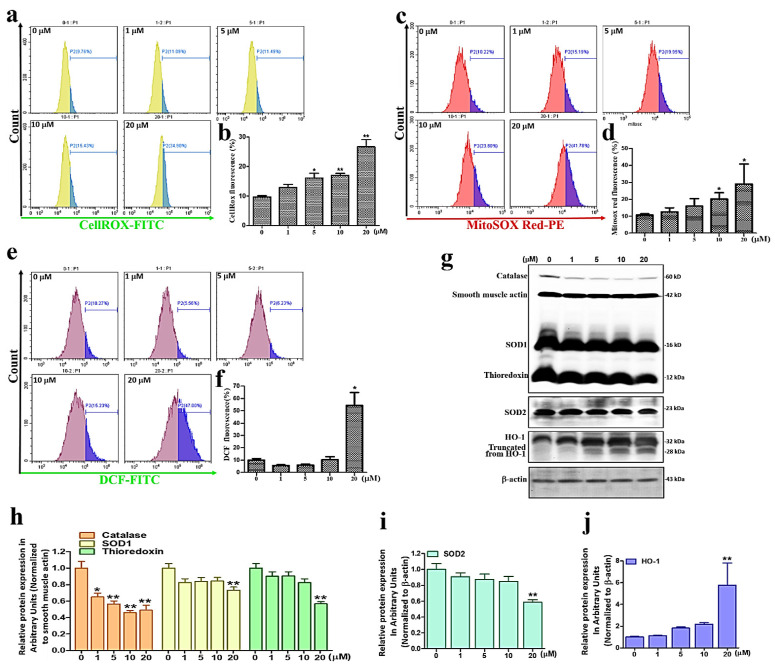
ROS and relevant protein expression levels measured after 24 h of sinularin treatment in U87 MG cells. (**a**) Detection of ROS levels in mitochondria and the nucleus using CellROX^TM^ Green. (**b**) Analysis of CellROX^TM^ Green signals. (**c**) Detection of mitochondrial O_2_^•−^ levels using MitoSOX^TM^ Red. (**d**) Analysis of MitoSOX^TM^ Red signals. (**e**) Detection of intracellular ROS levels in mitochondria using CM-H_2_DCFDA. (**f**) Analysis of DCF signals. (**g**) Western blot measurement of catalase, SOD1, SOD2, thioredoxin, and HO-1. Full, uncropped Western blot gel can be found on Supplementary File Figure S1b. (**h**) Analysis of catalase, SOD1, and thioredoxin. (**i**) Analysis of SOD2. (**j**) Analysis of HO-1. Each bar represents the mean ± standard error of the mean. * *p* < 0.05 and ** *p* < 0.01 relative to control.

Considering the ability of sinularin to induce ROS in cells [19], we next evaluated ROS levels using CellROX^TM^ Green, MitoSOX^TM^ Red, and CM-H_2_DCFDA staining methods following 24 h of sinularin treatment in U87 MG cells. CellROX^TM^ Green is used to detect ROS in mitochondria and the nucleus, while MitoSOX^TM^ Red identifies mitochondrial O_2_^•−^ levels and CM-H_2_DCFDA detects ROS levels intracellularly [17,19]. Our results showed that CellROX^TM^ Green signals were significantly elevated upon treatment with sinularin at 5 μM and above concentrations (Figure 3a,b), while MitoSOX^TM^ Red signals were significantly elevated with sinularin at 10 μM and above concentrations (Figure 3c,d). The signals for DCF (the oxidation product of CM-H_2_DCFDA) were significantly elevated with sinularin at 20 μM (Figure 3e,f). These results showed a concentration-dependent increase in ROS levels. We subsequently monitored the expression levels of the antioxidant proteins catalase, SOD1, SOD2, thioredoxin [20], and heme oxygenase 1 (HO-1) [21] (Figure 3g). Our findings demonstrate that the expression levels of catalase, SOD1, SOD2, and thioredoxin were significantly reduced following treatment with sinularin at 20 μM (Figure 3h,i). By contrast, HO-1 levels were significantly increased with sinularin at the same concentration (Figure 3j).

### 3.4. Sinularin Causes Dysfunction of Mitochondrial Respiration

**Figure 4:**
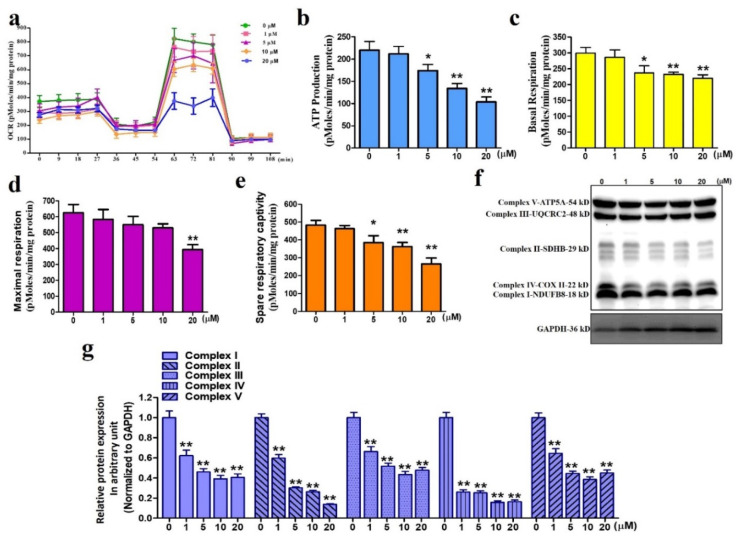
Changes in oxygen consumption rates and enzymatic complexes I to V of the OXPHOS electron transport chain. (**a**) Measurements of oxygen consumption rates. (**b**) Analysis of ATP production. (**c**) Analysis of basal respiration. (**d**) Analysis of maximal respiration. (**e**) Analysis of spare respiration capacity. (**f**) Western blot measurement of the enzymatic complexes I to V. Full, uncropped Western blot gel can be found on Supplementary File Figure S2a. (**g**) Analysis of the enzymatic complexes I to V. Each bar represents the mean ± standard error of the mean. * *p* < 0.05 and ** *p* < 0.01 relative to control.

Since mitochondria are the major site of ROS production, the oxygen consumption rates (OCRs) can serve as an indication of mitochondrial OXPHOS efficiency. We thus measured the OCRs by sequentially adding specific antagonists for the enzymatic complexes I to IV of the OXPHOS electron transport chain, particularly oligomycin, FCCP, and rotenone. The use of these antagonists allowed us to calculate the following mitochondrial respiration parameters: basal respiration, ATP production, maximal respiration, and spare respiration capacity. We evaluated sinularin-induced changes in the respiration of U87 MG cells after 24 h of treatment at 0, 1, 5, and 10 μM (Figure 4a). With 20 μM of sinularin, our results revealed that basal respiration, ATP production, maximal respiration, and spare respiration capacity were all significantly inhibited. Similarly, at lower sinularin concentrations (5 and 10 μM), mitochondrial respiration parameters, except for the maximal respiration, were significantly inhibited (Figure 4b–e). The enzymatic complexes I to V critical for the OXPHOS electron transport chain were also analyzed by Western blot analysis. The results showed significant activity inhibition in a dose-dependent manner (Figure 4f,g). These findings suggest that sinularin concentrations as low as 5 μM could significantly inhibit mitochondrial respiration. A subsequent study was conducted to measure changes in expression levels of the OXPHOS electron transport chain enzymatic complexes I to V. Our Western blot findings measuring the protein levels of these protein complexes indicate significant decreases with sinularin at 1 μM and above concentrations, suggesting that these changes could underlie the observed mitochondrial alterations.

### 3.5. Sinularin Interferes with Cell Migration and Angiogenesis

**Figure 5:**
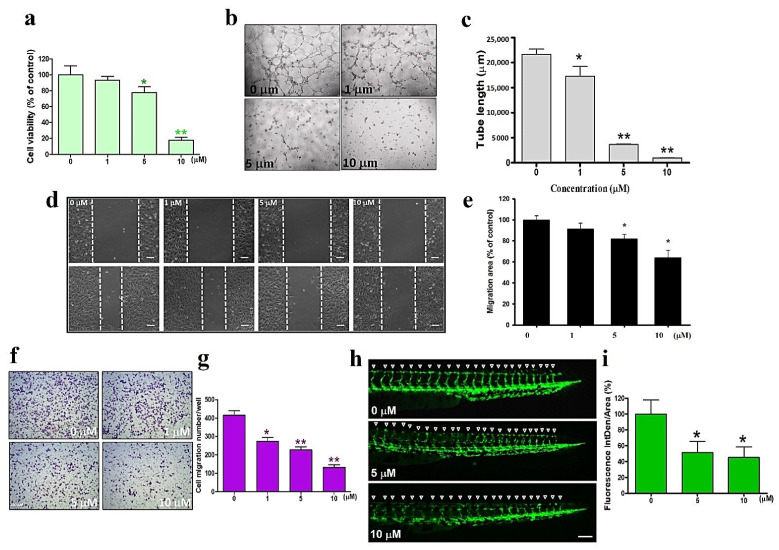
Antiangiogenic effects of sinularin in EA.hy926 cells and zebrafish. (**a**) Cell viability of EA.hy926 cells under sinularin treatment. (**b**) Tube length morphology on Matrigel after treatment with VEGF or control, with or without sinularin (0–10 μM) in EA.hy926 cells. (**c**) Quantitative results of tube length after treatment with VEGF or control, with or without sinularin (10 μM) in EA.hy926 cells. (**d**) The mobility in EA.hy926 cells was assessed using a wound healing assay. (**e**) Quantitative results of cell migration area. (**f**) Transwell analysis of EA.hy926 cells migration ability after sinularin treatment. (**g**) Analysis of sinularin-induced migration. (**h**) One day postfertilization, the angiofluorescent zebrafish eggs were soaked in Hank’s buffer containing 0, 5, or 10 μM of sinularin. Following two additional days in culture, zebrafish embryos were photographed under a fluorescence microscope, showing the formation of their intersegmental vessels. White triangles represent where intersegmental vessels are. Scale bar = 100 mm. (**i**) Quantitative results of fluorescent intersegmental vessels. Each bar represents the mean ± standard error of the mean. * *p* < 0.05 and ** *p* < 0.01 relative to control.

Since Ferrando et al. revealed that HO-1-regulation prevents angiogenesis [22], we next evaluated the antiangiogenic effects of sinularin using the fusion cell line EA.hy926. The viability of EA.hy926 cells was significantly inhibited by sinularin treatment for 24 h at 5 μM and above concentrations (Figure 5a). Moreover, sinularin at 10 μM reduced the VEGF-induced lengthening of vascular tubes in a concentration-dependent manner, as revealed using the tube formation assay with human umbilical vein endothelial cells (HUVEC) (Figure 5b,c). Using the wound healing assay, the mobility of EA.hy926 cells was also found to be altered by sinularin in a dose-dependent manner after 18 h, with a significant effect observed at 5 μM and above concentrations (Figure 5d,e). Likewise, using the transwell migration assay, the numbers of migrated EA.hy926 cells were reduced by sinularin in a dose-dependent manner, with a significant effect observed at 1 μM and above (Figure 5f,g). A zebrafish model was next used to investigate vessel formation after sinularin treatment. We assessed the effects of sinularin at 0, 5, and 10 μM on the formation of intersegmental vessels (ISVs) in transgenic Tg(fli1:EGFP) angiofluorescent zebrafish larvae. Our results demonstrate that sinularin inhibits the angiogenesis of intersegmental blood vessels in zebrafish larvae (Figure 5h,i).

### 3.6. Sinularin Inhibits Angiogenesis by Downregulating eNOS and VEGF, and Upregulating HO-1

**Figure 6:**
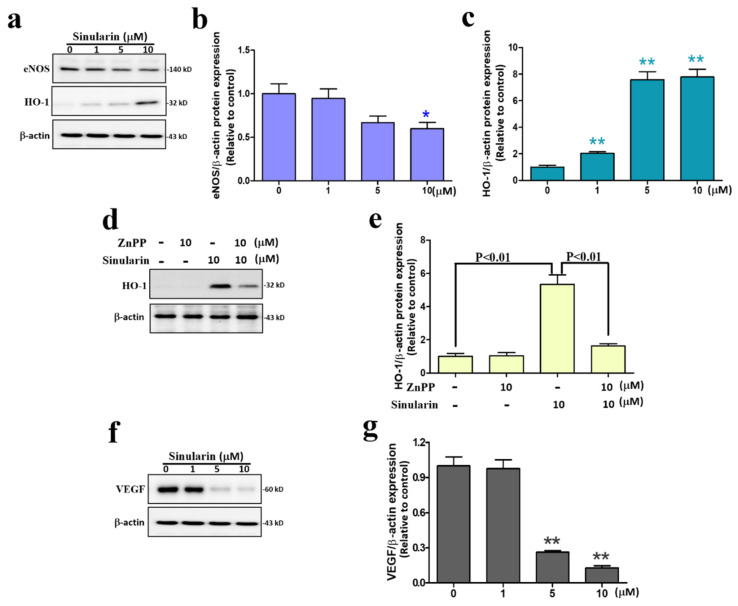
Anti-angiogenesis effects of sinularin on eNOS, HO-1, and VEGF in EA.hy926 cells. (**a**) Western blotting measuring eNOS and HO-1, and the internal control β-actin. Full, uncropped Western blot gel can be found on Supplementary File Figure S2b. (**b**) Quantification of eNOS versus β-actin. (**c**) Quantification of HO-1 versus β-actin. (**d**) Western blotting measuring HO-1 after treatment with or without sinularin and/or zinc protoporphyrin (ZnPP). Full, uncropped Western blot gel can be found on Supplementary File Figure S2c. (**e**) Quantification of HO-1 versus β-actin. (**f**) Western blotting measuring VEGF after treatment with or without sinularin. Full, uncropped Western blot gel can be found on Supplementary File Figure S2d. (**g**) Quantification of VEGF versus β-actin. Each bar represents the mean ± standard error of the mean. * *p* < 0.05 and ** *p* < 0.01 relative to control.

The strong effect of sinularin at preventing angiogenesis in human endothelium cells and zebrafish prompted further investigation of the expression levels of angiogenic factors such as endothelial nitric oxide synthase (eNOS) in addition to HO-1 in EA.hy926 cells. Our Western blot results showed that the expression levels of eNOS decreased while those of HO-1 increased in EA.hy926 cells after 24 h of treatment with sinularin (Figure 6a–c). Subsequently, we pretreated the cells with a HO-1 inhibitor, zinc protoporphyrin (ZnPP). Our findings indicate that the sinularin-induced upregulation of HO-1 was significantly dampened by ZnPP, further supporting the ability of sinularin to increase HO-1 expression (Figure 6d,e). We also found that VEGF expression levels were significantly decreased by sinularin at 5 μM and above concentrations (Figure 6f,g).

## 4. Discussion

**Figure 7:**
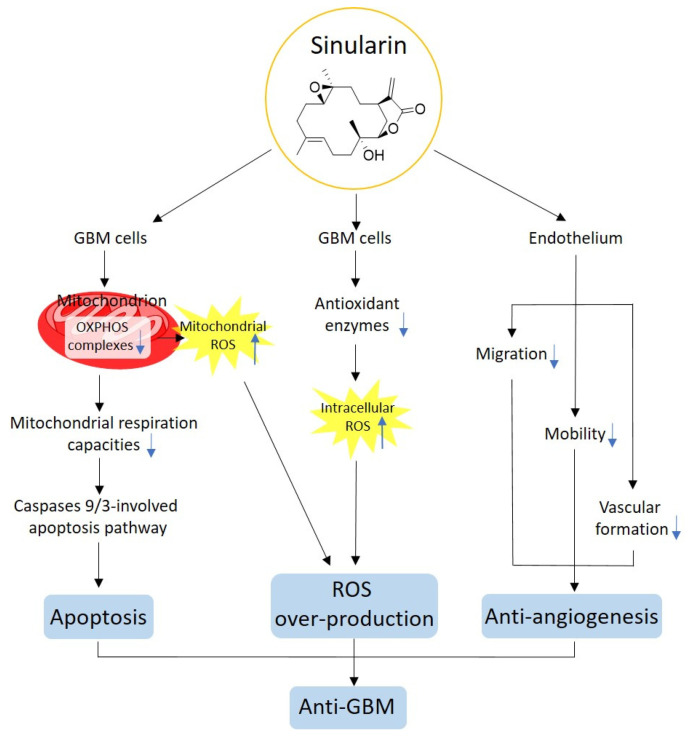
Proposed anti-cancer mechanisms of sinularin. GBM, glioblastoma multiforme; OXPHOS, oxidative phosphorylation; ROS, reactive oxygen species.

Based on the previous literature and our current findings, the anti-cancer mechanisms of sinularin are summarized in Figure 7. In particular, sinularin compromises mitochondrial respiration by downregulating the activities of OXPHOS complexes, which in turn initiates caspase-dependent apoptosis. The downregulated OXPHOS complexes lead to increased mitochondrial ROS. These intracellular ROS levels are increased due to the sinularin-induced inhibition of antioxidant enzymes. Both mitochondrial and intracellular ROS exacerbate oxidative stress. The sinularin-induced antiangiogenesis results from an inhibition of cell migration, mobility, and vascular tube formation. Taken together, these findings indicate that sinularin demonstrates anti-cancer effects through the induction of apoptosis, ROS, and antiangiogenesis in GBM cells.

Prior to this study, sinularin has been studied for its anti-cancer effects in various cancer types. Chang et al. found that sinularin inhibits oral cancer cells through the induction of oxidative stress-mediated G2/M arrest and apoptosis [16]. Chung et al. reported that sinularin causes DNA damage, G2/M phase arrest, and apoptosis in human hepatocellular carcinoma cells [15]. Huang et al. revealed that sinularin similarly inhibits breast cancer cells via G2/M arrest, apoptosis, and oxidative DNA damage [14]. Ma et al. also showed that sinularin exerts anti-tumor effects in renal cancer cells dependent on ROS overproduction [23]. Wu et al. further indicated that sinularin-induced apoptosis results from mitochondrial dysfunction and inactivation of the pI3K/Akt/mTOR pathway in gastric cancer cells [13]. These prior studies focused on investigating apoptosis as the mode of cell death induced by sinularin in cancer cells. However, oxidative stress was also shown to be increased by sinularin in these previous studies, implicating apoptosis but also oxidative stress as the main anti-cancer effects of sinularin. In the current study, we indeed observed sinularin-induced apoptotic cell death and ROS overproduction in GBM cells.

In addition to the well-known apoptotic property of sinularin, we found an intriguing anti-angiogenesis outcome associated with HO-1 upregulation, which was not yet described in the literature. Our Western blot analysis showed that the alteration in the HO-1 expression pattern was contrary to the other antioxidant enzymes evaluated, although HO-1 is primarily considered an antioxidant [21]. This suggested a different role for HO-1 in this cancer type. Since Ferrando et al. reported that HO-1 upregulation and downregulation of VEGF together prevented angiogenesis [22], we evaluated the effects of sinularin in this regard. Similar to Ferrando et al., our findings showed that sinularin-induced an upregulation of HO-1, ultimately leading to antiangiogenesis, as evidenced by the cellular and in vivo studies that are discussed below.

Antiangiogenesis is considered a main strategy to inhibit cancer, because angiogenesis is a critical step in cancer progression, which involves the activation, proliferation, tube formation, and migration of endothelial cells [24]. EA.hy926 is a cell line fused from HUVEC and a thioguanine-resistant clone of human lung adenocarcinoma A549 cells. This fusion cell line is commonly used in studies investigating angiogenesis [25,26,27]. For example, the studies conducted by Dredge et al. and Yang et al. used EA.hy926 cells to evaluate the inhibitory effects of test substances on angiogenesis [26,27]. Accordingly, the current study used the EA.hy926 cell line as a cellular testing platform for antiangiogenesis. In addition, multiple in vivo models have been developed to study antiangiogenic effects, including the chick chorioallantoic membrane assay, the zebrafish, mouse corneal angiogenesis assay, the mouse dorsal air sac model, and the mouse Matrigel plug test [28]. Among them, the zebrafish (Danio rerio) model has the advantages of high fertility and easy breeding, as well as transparent and favorable observation in the early stages of embryonic development. The development process of zebrafish has also many similarities to that of mammals, making it an excellent model for angiogenesis investigations.

In the current study, the antiangiogenesis effects of sinularin were not only evidenced in cells but also in an animal model, the zebrafish. Initially, we used EA.hy926 cells to evaluate the antiangiogenic effects of sinularin. Our in vitro findings showed antiangiogenic properties of sinularin such as cell death, but also a prevention of tube formation, cell migration, and cell mobility. Subsequently, we used transgenic vascular fluorescent zebrafish Tg(fli1:EGFP) as an animal model to confirm the effects in vivo. In this animal model, all the vascular endothelial cells express the enhanced green fluorescent protein. This model relies on the developmental process of zebrafish embryos, which enter the angiogenic stage after initial formation of the primitive vasculature. As in other vertebrates, the ISV of the trunk is one of the first vessels formed during angiogenesis [29]. Another relevant fact is that the circulatory system of zebrafish begins to emerge 24–26 h after fertilization, while the ISV can be observed in the fish body trunk from the lower dorsal aorta and posterior cardinal vein at about 1.5 day postfertilization [30]. Therefore, in this study, sinularin was applied at 1-day postfertilization and the formation of intersegmental blood vessels was observed at 2 days postfertilization. Our zebrafish results showed strong antiangiogenesis effects of sinularin in agreement with our findings in EA.hy926 cells. At the molecular level, we found that the expression levels of eNOS and VEGF were decreased, while HO-1 was increased by sinularin. Excessive expression of eNOS and VEGF is well-known to promote angiogenesis [9,31]. Thus, in addition to HO-1 upregulation, the decreases in these two proteins further supported the sinularin-induced antiangiogenesis effects. Nevertheless, HO-1 was reported to have a complex role in cancer progression depending on its location. Intracellularly, HO-1 expression shows antioxidant, anti-apoptotic, and cytoprotective properties, whereas extracellular HO-1 affects the tumor microenvironment by facilitating angiogenesis and metastasis. However, this finding may not apply to all cancer types. Hoang et al. have indicated in their review that the complex roles of HO-1 are TME- and cell type-dependent [28]. Our findings further support a complex involvement of HO-1 in cancer progression.

Intriguingly, antibrain cancer drugs with anti-inflammatory properties may provide treatment for GBM. A previous meta-analysis report indicated that the use of nonsteroidal anti-inflammatory drugs (NSAIDs) is associated with a significantly lower risk of developing GBM, while the correlation between NSAIDs use and GBM tumor risk is dose-dependent [32]. At the molecular level, cyclooxygenase-2 (COX-2), a key mediator of inflammatory pathways, has been widely reported to be overactive in brain tumors, and may contribute to the correlation between NSAIDs use and reduced GBM tumor risk [33]. Previous studies suggest that inhibitors that can attenuate inflammation by downregulating COX-2 activity may also show anti-GBM effects. In our previous study, sinularin demonstrated anti-inflammatory properties in a carrageenan-induced inflammatory rat model [34], partially by abrogating COX-2 activity. It is therefore plausible that sinularin inhibits COX-2, conferring an advantage for the treatment of GBM, although further research is needed.

Based on chemical properties prediction, sinularin would have a molecular weight (MW) of 334.21, a polar surface area (PSA) of 59.06 Å, a distribution coefficient (LogD) of 3.67 at pH 7.4, a partition coefficient (LogP) of 3.33, a total number of nitrogen and oxygen (N + O) of 4, and a hydrogen bond donor. These properties may allow sinularin to pass through the blood–brain barrier (BBB), as these predicted values are approximate to Trippier and Good’s statistically summarized “drug-like” properties for central nervous system diseases as follows: (1) (N + O) < 6; (2) PSA < 70 Å; (3) MW < 450; (4) LogD = 1–3; (5) cLogP–(N + O) ≥ 0; and (6) without hydrogen bond donors [35]. The potential BBB-penetratable property of sinularin, which warrants further investigation, could serve as an additional advantage for antibrain cancer drug development. Together with the clearly demonstrated anti-cancer properties evidenced in this study, the potential BBB-penetratable property of sinularin could make it a promising candidate for further development in GBM treatment.

Despite promising anti-apoptosis and -angiogenesis results shown in this study, there are other limitations that are also needed to be further investigated. Firstly, patient-derived GBM cells contain a small population of stem (-like) GBM cells that present distinct characteristics from non-stem (-like) GBM cells, such as self-renewal and tumor cell heterogeneity. The stemness is believed to cause chemoresistance [36]. Whether sinularin can exert cytotoxic effects on the cancer stem cells remain to be revealed, even though some of the established GBM clones (such as U87 MG) used in this study are reported to express stem-like biomarkers [37]. This is because they were cultured in defined conditions that are not reflective to clinical conditions. Another limitation is the tumor microenvironment (TME), which is difficult to mimic in the cultured room; hence, little is known about sinularin’s anti-neoplastic effects on the TME. In addition, clinical findings indicate that radioresistance renders brain tumors elusive, and it is believed that the four Rs of radiology (DNA damage repair, reoxygenation, redistribution of the cell cycle, and repopulation) are the underlying mechanisms of radioresistance [38]. Based on our findings in this study, sinularin could partially overcome radioresistance since it is able to induce DNA damage and the induction of ROS. Nevertheless, additional studies are required to verify the effect.

In addition to sinularin, other natural products and their analogues warrant investigation in the aspect of treating GBM, as many studies have shown add-on anti-cancer effects when co-administering with the standard GBM drug TMZ. For example, quercetin isolated from *Allium cepa* increased the chemosensitivity of TMZ in U87 and U251 GBM cell lines [39]. *Vitis vinifera*-derived resveratrol-induced ROS generation, decreased antiapoptotic protein Bcl-2, and enhanced TMZ sensitivity in SHG44 GBM cells [40]. Moreover, the hydroalcoholic extract of *Zataria multiflora* enhanced the radiosensitivity of A172 GBM cells [41]. These studies implicate that natural products could both enhance chemo- and radio-sensitivity, giving the drug development community a source for developing new therapies against GBM.

## 5. Conclusions

In this study, sinularin has displayed cytotoxicity against the GBM cells, showing a consistent mode of cell death with prior studies. More intriguingly, this study has found that sinularin possesses anti-angiogenesis characteristics, as evidenced by the zebrafish model and cellular platform, which further places importance on sinularin in the area of anti-cancer drug development.

## Data Availability

The data is contained within this article and supplementary files.

## Supplementary Materials

The following supporting information can be downloaded at: https://www.mdpi.com/article/10.3390/antiox11081433/s1, Figure S1: Original, uncropped images of the Western blots for Figure 2d and Figure 3g displayed in the text and results; Figure S2: Original, uncropped images of the western blots for Figure 4f, Figure 5f, and Figure 6f displayed in the text and results; Table S1: Information on primary antibodies used in the Western blot analysis of this study.
