# Supplementary material for: Sinularin Induces Oxidative Stress-Mediated Apoptosis and Mitochondrial Dysfunction, and Inhibits Angiogenesis in Glioblastoma Cells
Source: Antioxidants (Basel). 2022 Jul 23;11(8):1433. doi: 10.3390/antiox11081433 (PMC9394238; doi:10.3390/antiox11081433)
Supplement: Supplementary file 1 [file antioxidants-11-01433-s001.zip › Supplementary File.pdf]

Sinularin induces oxidative stress-mediated apoptosis and mitochondrial dysfunction, and inhibits angiogenesis in human brain glioblastoma multiforme

Shih-Yuan Hsu[1,2]†, Zhi-Hong Wen[1]†, Po-Chang Shih[1,6], Hsiao-Mei Kuo[1,3], Sung-Chun Lin[4], Hsin-Tzu Liu[5], Yi-Hsin Lee[1], Yi-Jen Wang[1], Wu-Fu Chen[1,6], Nan-Fu Chen[7,8,9]*

[1] Department of Marine Biotechnology and Resources, National Sun Yat-Sen University, Kaohsiung 80424, Taiwan; 07077@ptch.org.tw (S.-Y.H.); wzh@mail.nsysu.edu.tw (Z.-H.W.); hsiaomeikuo@gmail.com (H.-M.K.); po-chang.shih.14@ucl.ac.uk (P.-C.S.); lesinsin@gmail.com (Y.-H.L.); yijenwang0217@gmail.com (Y.-J W); ma4949@cgmh.org.tw (W.-F.C)

[2] Department of neurosurgery, Pingtung Christian Hospital, Pingtung 90059, Taiwan; 07077@ptch.org.tw (S.-Y.H.)

[3] Center for Neuroscience, National Sun Yat-sen University, Kaohsiung 80424, Taiwan;

[4] Department of Orthopedic Surgery, Pingtung Christian Hospital, Pingtung 90059, Taiwan; linsungchun@yahoo.com.tw (S.-C.L.)

[5] Department of Medical Research, Hualien Tzu Chi Hospital, Buddhist Tzu Chi Medical Foundation, Hualien 970473, Taiwan; hsintzuliu@tzuchi.com.tw

[6] Department of Neurosurgery, Kaohsiung Chang Gung Memorial Hospital and Chang Gung University College of Medicine, Kaohsiung 83301, Taiwan; ma4949@cgmh.org.tw (W.-F.C)

[7] Division of Neurosurgery, Department of Surgery, Kaohsiung Armed Forces General Hospital, Kaohsiung 80284, Taiwan; chen06688@gmail.com (N.-F.C)

[8] Institute of Medical Science and Technology, National Sun Yat-sen University, Kaohsiung 804201, Taiwan; chen06688@gmail.com (N.-F.C)

[9] Center for General Education, Cheng Shiu University, Kaohsiung 833301, Taiwan; chen06688@gmail.com (N.-F.C)

† These authors contributed equally to this work.

* Correspondence: chen06688@gmail.com (N.-F.C)

TEL: +886-7-7494963; Fax: +886-7-7498281

1. Supplementary Figures

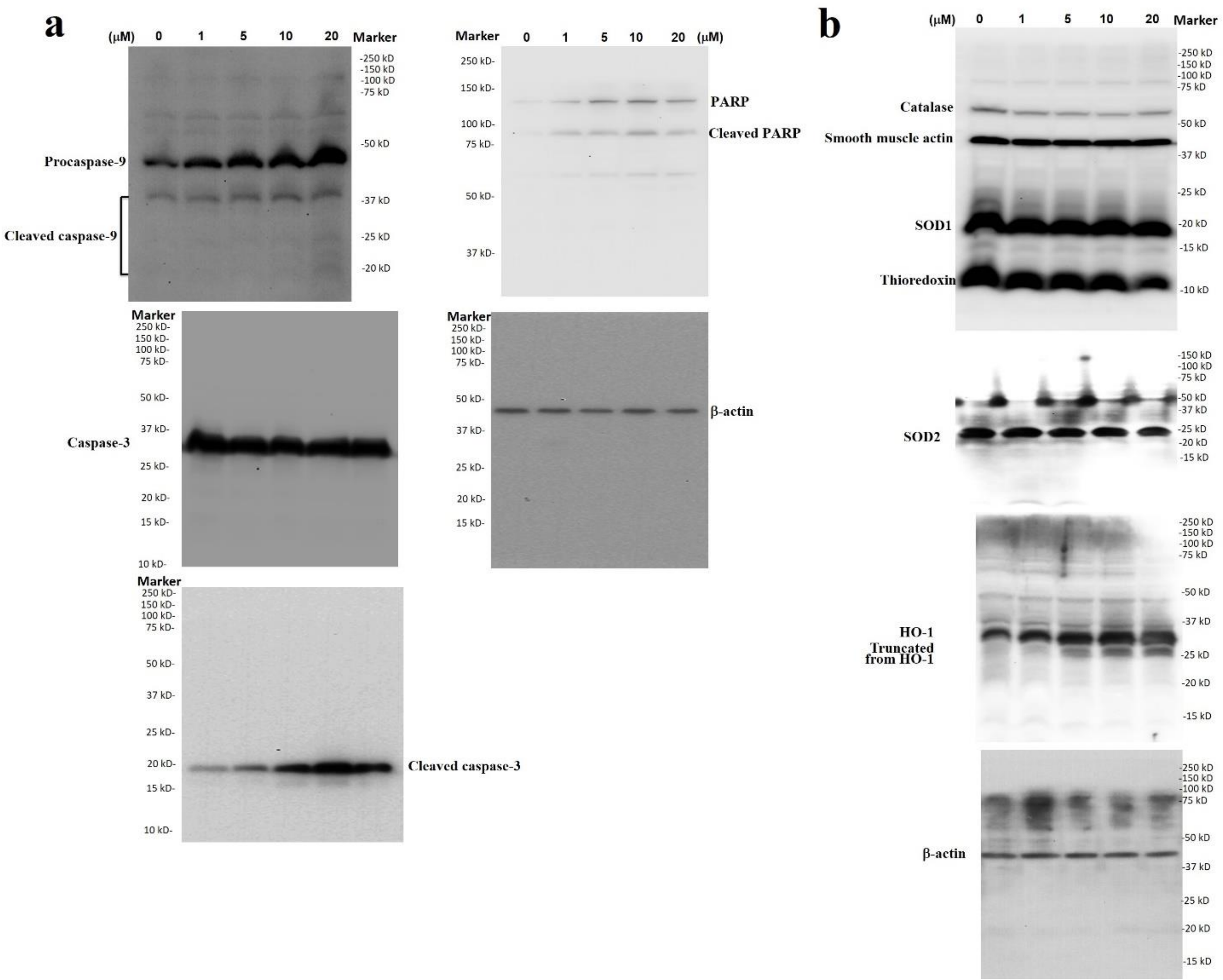

Supplementary Figure S1. Original, uncropped images of the western blots for Fig. 2D, 3G displayed in the text and results: (**a**) The bands of the cleaved caspase-9, pro-caspase-9, cleaved caspase-3, pro-caspase-3, PARP, cleaved PARP and β-actin and their expected molecular weight; (**b**) The bands of catalase, smooth muscle actin, SOD1, thioredoxin, SOD2, HO-1, truncated HO-1 and β-actin and their expected molecular weight. β-actin and smooth muscle actin were used as the protein loading controls.

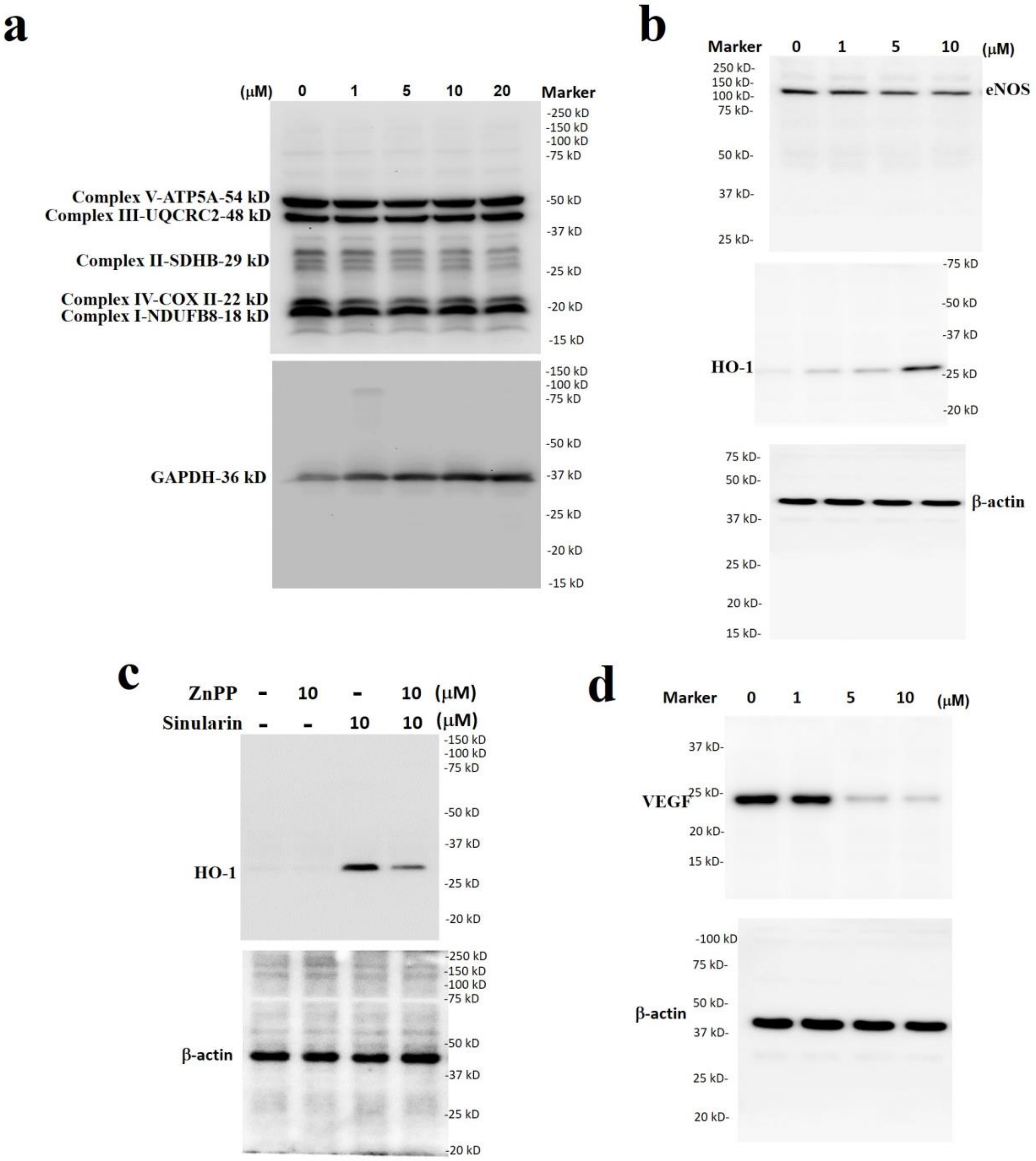

Supplementary Figure S2. Original, uncropped images of the western blots for Fig. 4F, 5F, and 6F displayed in the text and results: (**a**) The bands of the mitochondrial complexes I-V and GADPH and their expected molecular weight; (**b**) The bands of eNOS, HO-1, and β-actin; (**c**) The bands of HO-1 and β-actin; (**d**) The bands of VEGF and β-actin. GADPH and β-actin were used as the protein loading controls.

2. Supplementary Table

Table S1. Information on primary antibodies used in the western blot analysis of this study.

Antigen	Host	Catalogue	Clonality	Supplier	Dilution ratio
Anti-caspase 3	Rabbit	14420	Polyclonal	Cell Signaling	1:1000
Cleaved caspase 3	Rabbit	9664S	Monoclonal	Cell Signaling	1:1000
Anti-caspase 9	Rabbit	9502	Polyclonal	Cell Signaling	1:1000
Cleaved caspase 9	Rabbit	9502	Monoclonal	Cell Signaling	1:1000
PARP	Rabbit	9542S	Polyclonal	Cell Signaling	1:1000
Cleaved PARP	Rabbit	9541	Polyclonal	Cell Signaling	1:1000
Total OXPHOS Human WB Antibody Cocktail	Mouse	ab110411	Monoclonal	Abcam	1:1000
Anti-SDHB	Mouse	ab14714	Monoclonal	Abcam	1:1000
Anti-UQCRC2	Mouse	ab14745	Monoclonal	Abcam	1:1000
Anti-ATP5A	Mouse	ab14748	Monoclonal	Abcam	1:1000
Anti-COX II	Mouse	ab14745	Monoclonal	Abcam	1:1000
Anti-NDUFB8	Mouse	ab14748	Monoclonal	Abcam	1:1000
Oxidative Stress Defense (Catalase, SOD1, TRX, smooth muscle Actin) Western Blot Cocktail	Rabbit	ab179843	Monoclonal	Abcam	1:1000
Anti-catalase	Rabbit	ab179843	Monoclonal	Abcam	1:1000
Anti-SOD1	Rabbit	ab179843	Monoclonal	Abcam	1:1000
Anti-thioredoxin	Rabbit	ab179843	Monoclonal	Abcam	1:1000

Anti-smooth muscle actin	Rabbit	ab179843	Monoclonal	Abcam	1:1000
Anti-SOD2	Rabbit	ab68155	Monoclonal	Abcam	1:1000
eNOS	Mouse	sc-376751	Monoclonal	Santa Cruz	1:1000
HO-1	Mouse	sc-136960	Monoclonal	Santa Cruz	1:1000
VEGF	Mouse	sc-7269	Monoclonal	Santa Cruz	1:1000
Anti-β-actin	Mouse	A5441	Monoclonal	Sigma-Aldrich	1:5000
