# Supplementary figures and images for: Sinularin Induces Oxidative Stress-Mediated Apoptosis and Mitochondrial Dysfunction, and Inhibits Angiogenesis in Glioblastoma Cells
Source: Antioxidants (Basel). 2022 Jul 23;11(8):1433. doi: 10.3390/antiox11081433 (PMC9394238; doi:10.3390/antiox11081433)

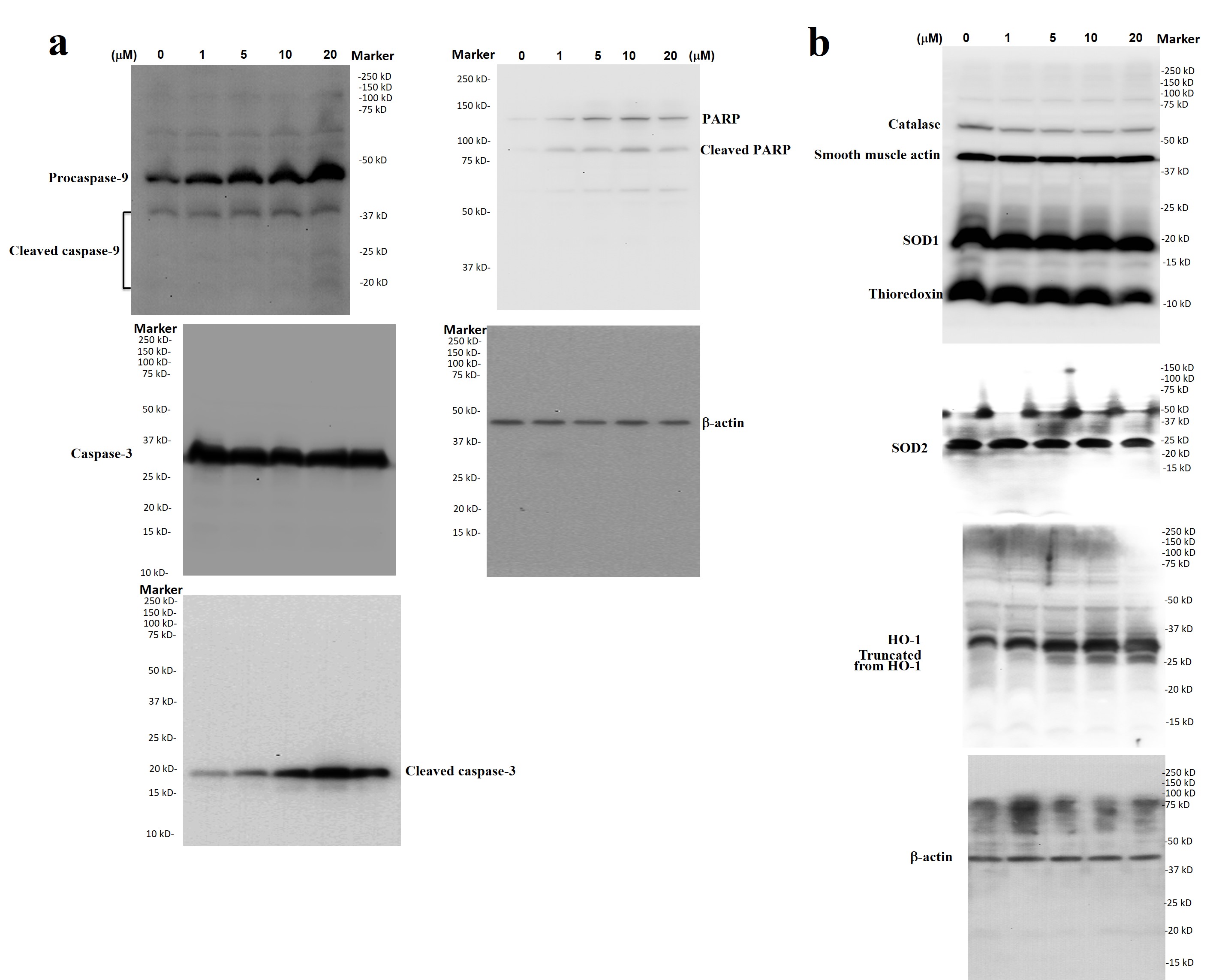

Supplement: Supplementary file 1 [file antioxidants-11-01433-s001.zip › SuppFig1.jpg]

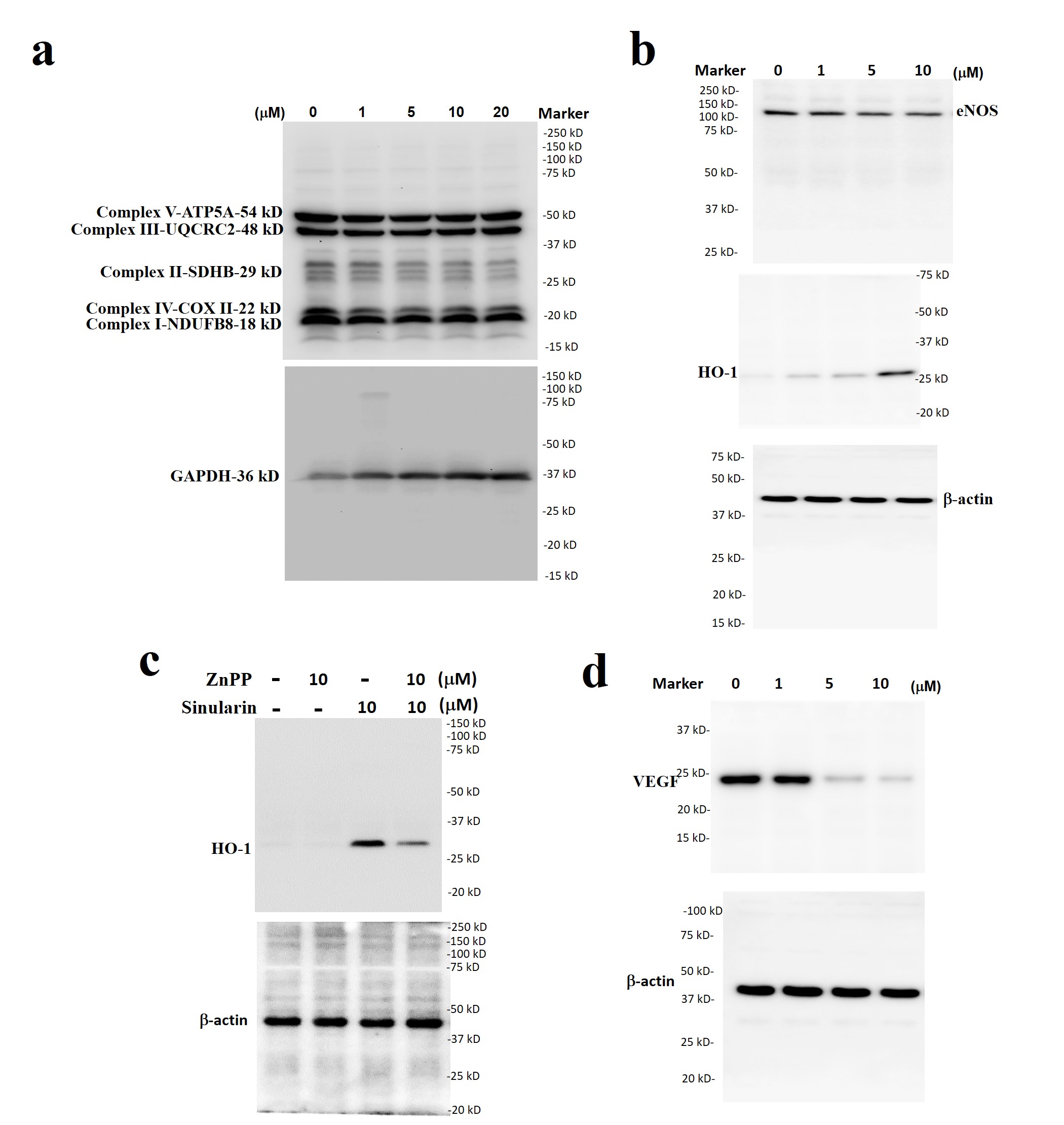

Supplement: Supplementary file 1 [file antioxidants-11-01433-s001.zip › SuppFig2.jpg]
